# The acceptability of using wearable electronic devices to monitor physical activity of patients with Multiple Myeloma undergoing treatment: a systematic review.

**DOI:** 10.46989/001c.121406

**Published:** 2024-07-29

**Authors:** Tommy Brown, Ann Muls, Charlotte Pawlyn, Kevin Boyd, Susanne Cruickshank

**Affiliations:** 1 Haematology Research Royal Marsden NHS Foundation Trust; 2 Royal Marsden NHS Foundation Trust; 3 Institute of Cancer Research

**Keywords:** systematic review, Multiple Myeloma, wearable devices, physical activity, haemato-oncology, haematology, wearables

## Abstract

**Introduction:**

Multiple myeloma (MM) is diagnosed in 6,000 people in the UK yearly. A performance status measure, based on the patients’ reported level of physical activity, is used to assess patients’ fitness for treatment. This systematic review aims to explore the current evidence for the acceptability of using wearable devices in patients treated for MM to measure physical activity directly.

**Methods:**

Three databases were searched (MEDLINE, EMBASE and CINAHL) up until 7th September 2023. Prospective studies using wearable devices to monitor physical activity in patients on treatment for MM were included. Bias across the studies was assessed using the CASP tool.

**Results:**

Nine studies, with 220 patients on treatment for MM, were included. Only two studies had a low risk of bias. Different wearable device brands were used for varying lengths of time and were worn on either the wrist, upper arm, or chest. Adherence, reported in seven studies, ranged from 50% to 90%. Six studies reported an adherence greater than 75%. Although physical activity was also measured in a heterogenous manner, most studies reported reduced physical activity during treatment, associated with a higher symptom burden.

**Conclusion:**

Monitoring patients receiving treatment for MM with a wearable device appears acceptable as an objective measure to evaluate physical activity. Due to the heterogeneity of the methods used, the generalisability of the results is limited. Future studies should explore the data collected prospectively and their ability to predict relevant clinical outcomes.

## Introduction

Multiple myeloma (MM) is a malignancy of plasma cells, with around 6,000 new patients being diagnosed each year in the UK, representing about 2% of all new cancer cases.^1^ Over the last 50 years, life expectancy has improved vastly, with the 10-year survival rate increasing from 6.4% in the 1970s to 32.5%.^1^ This has been propelled using autologous stem cell transplants (ASCT), novel therapeutic treatments and enhanced supportive care.^2^

The use of wearable devices for the purpose of patient health monitoring within the context of healthcare has risen steeply over the last few years, and the advancements in wearable technology have been considerable, with the arrival of more advanced sensors, algorithm–based technology, and artificial intelligence.^3^ In this context, a wearable device is an electronic device that uses sensors to remotely collect real-time health-related data,^4^ and is often referred to as a wearable fitness device or fitness tracker, but throughout this paper will be referred to as wearable device. Information can be collected on numerous data points, including physical activity, heart rate, oxygen saturation, and sleep quality.^5^ These data can be used alone or in combination with other information such as patient-reported outcomes, physical activity, performance status, and clinical outcomes. Data can be recorded and transferred wirelessly onto a database, offering a level of clinical information far superior to standard forms of paper-based activity assessment.

MM is associated with the highest symptom burden and lowest cancer-related quality of life (CR-QOL) among patients with haematological malignancies.^6^ Symptoms of disease can include painful bone lesions and limited levels of activity and physical movement. New treatments mean that MM patients are living longer; however, reduced quality life and symptom burden remain a significant issue. CR-QOL in MM varies, with the greatest burden occurring at diagnosis and relapse.^7^ Therefore, the monitoring of subjective data such as patient-reported outcomes and CR-QOL alongside objective data such as those from a wearable device, provides an opportunity to enhance knowledge to better understand our patients’ needs and predict their outcomes. Wearable devices enable patients to monitor their own health, empowering them by providing real-time data about their health, which can lead to better self-management and a sense of control over their condition.^8^ Continuous monitoring can help in the early detection of potential issues, allowing for timely intervention, which can provide peace of mind to patients.^9^ Wearable devices can also facilitate better communication between patients and their healthcare providers by providing consistent health-related information in a timely manner.^10^ Wearable technology in MM therefore has the potential to offer new ways of understanding the performance status and overall health condition of patients, which in turn may help improve their management and treatment plans to allow an informed, personalised, and adaptable approach to their care.^11^ However, while continuous monitoring using wearable devices can offer significant benefits for patients with MM, it is important to note that there is also the potential for increased anxiety and stress. The continuous access to health data can be overwhelming for some patients, leading to unnecessary worry or misinterpretation of information.^12^ Patients might feel pressure to maintain certain health metrics, or the wearable devices might show data which seems alarming but is clinically insignificant, all leading to unnecessary stress and, potentially, unnecessary consultations with their medical team.^13^

In practice, healthcare professionals assess patient’s fitness using a performance status (PS) assessment, which is usually based on a patient’s perceived or reported level of activity and physical ability.^14^ The most common used are Eastern Cooperative Oncology Group (ECOG) and the Karnofsky Performance Scale (KPS). However, these scales have substantial limitations, including large variability in scoring between healthcare professionals.^15^ One study^16^ compared healthcare professional assessments with objective measurements and noted that 80% of patients assessed as an ECOG PS 0-1, should have been assessed as ECOG PS 3 (>50% of waking hours resting), based on objective measurements. The variation with ECOG suggests that a more comprehensive assessment of physical activity function is required.

Myeloma-specific frailty scores, such as the International Myeloma Working Group (IMWG) frailty score^17^ and the UK Myeloma Research Alliance (UKMRA) Myeloma Risk Profile (MRP), are specialised tools also utilised in clinical practice to assess the functional status and overall health of patients with MM.^18^ These scoring systems integrate various parameters including age, comorbidities, and performance status to stratify patients into risk categories, aiding clinicians to make individualised treatment decisions and prognostication. However, the main limitations of these scoring systems are the time that they require to administer in the clinic and their inherent subjectivity. Therefore, the use of an objective physical activity measure using “real-time” data in conjunction with these scoring systems would enhance decision making. Continuous passive monitoring using wearable devices could objectively gather levels of activity over long periods of time, reducing potential reporting bias and could inform on disease status and tolerability of emerging therapies.^19^

Only a few studies offer evidence that demonstrates how wearable technology can improve a patient’s cancer management.^20^ However, wearable devices have the potential to transform healthcare by providing consistent health-related information in a timely manner. Although data demonstrating the positive impact of wearable technology in various areas of cancer care such as breast and lung cancer are available,^21^ there is limited evidence in MM. With advances in science, and an increased range of novel therapies currently being used in clinical trials, treatment options for patients with MM will change further over the next 5 to 10 years. This could affect how patients are managed, including a reduction in the use of standard treatments such as stem cell transplants. Levels of fitness to proceed or not with treatment are integral to the decision-making in MM, and gaining a more accurate picture using wearable devices and their impact on clinical outcomes or patient management must be explored fully.^22^ Closely assessing factors associated with patient outcomes, adherence, acceptability by patients’ valid data measures etc, will ensure that wearable devices are introduced into clinical practice, with the patient’s perspective integrated.

The aim of this review is to explore the current published evidence for acceptability of using wearable devices to assess physical activity in patients treated for MM.

## Materials and Methods

This systematic review used the Preferred Reporting Items for Systematic Reviews and Meta-Analyses (PRISMA) guidelines to ensure transparent and complete reporting.^23^ For this purpose, the Gough et al. (2017)^24^ and Booth et al. (2016)^25^ guidelines for systematic reviews were followed.

### Data sources

**Figure 1.**
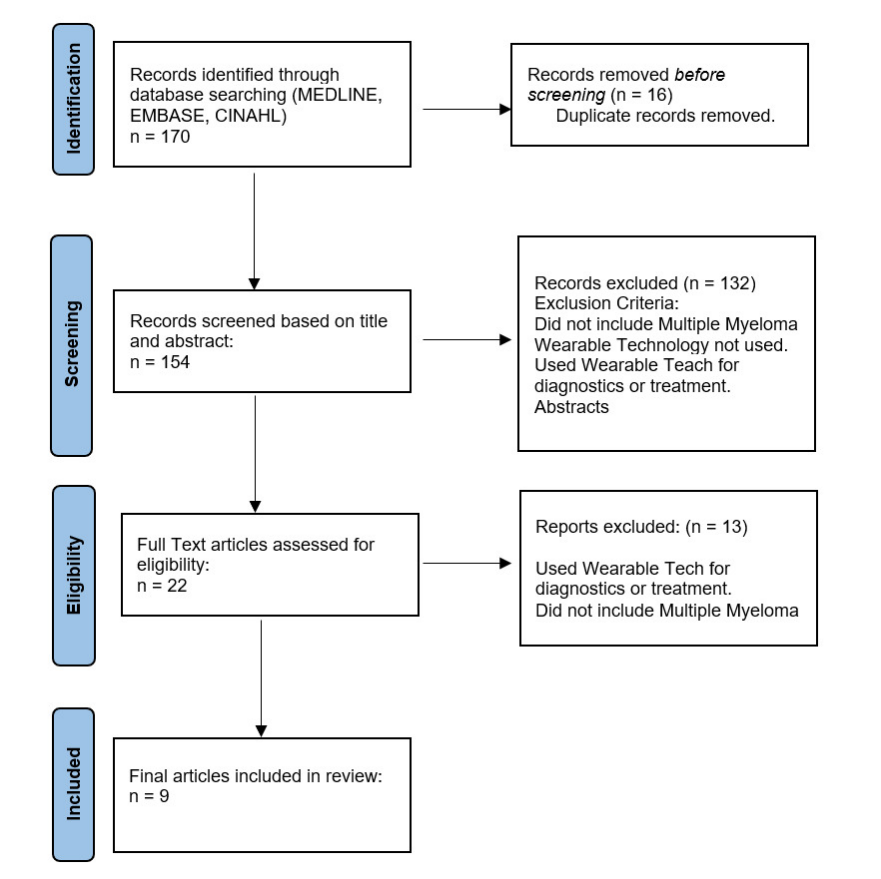
Flow Chart – Identification, Screening, and eligible papers

Relevant studies were identified by the author searching the following databases: MEDLINE, EMBASE and CINAHL. These were searched on 7^th^ September 2023, with no limitation in the date of publication. The main search terms included ‘myeloma’ “AND” “OR” ‘wearable’ (Appendix 1). Searching the PROSPERO database and Cochrane Library did not identify any existing similar systematic reviews. Additional resources on the PubMed database, such as “find similar search” and reference lists, were used to identify relevant studies. To ensure that the search was comprehensive, and studies had not been missed or wrongly excluded, general search engines (Google, Yahoo), and reference lists of included papers were checked. Specific exclusion criteria for the studies found through all these searches are described in Figure 1.

### Study eligibility

The inclusion criteria for studies were determined by following the components in the PEO(S) framework: the patient population (P) – Patients treated for MM; the exposure (E) – wearable devices; the outcome of the study (O) – patient adherence; and the study design (S) – prospective, observational, cohort studies using wearable devices to monitor performance status.

For eligibility, studies had to be written in English and include patients 18 years or older who had a confirmed diagnosis of MM. This review included prospective, pilot, feasibility, observational studies or (non-) randomised controlled trials. Studies that utilised a wearable electronic device with an objective measure and analysed physical activity were included, as well as stand-alone projects or projects nested within a larger study.

Studies were excluded if they did not include patients with MM or did not use a wearable device. Papers that only had a qualitative design (and no use of a wearable electronic device), protocol descriptions, systematic reviews, conference abstracts, case reports or editorials, or where the full text was not available were excluded.

### Study selection, Quality & Risk of bias Assessment

Studies found using the search strategy were screened by title and abstract, with included studies having their full text reviewed for eligibility. The quality and risk of bias in included studies was appraised independently by two reviewers (TB and AM) using a risk of bias assessment tool based on the CASP tool for cohort studies^26^ and previously published systematic review literature.^27–29^

Risk of bias components formed a 12-point list where, if a study provided sufficient information to meet the component, a score of one was given. Alternatively, a score of zero was given if a study did not provide sufficient information to meet the component. Discrepancies regarding the assessment were reviewed and, if required, were discussed with a third reviewer (SC). A total risk of bias score was calculated for each of the included study by dividing the total score by 12 (the total number of items) and presented as a percentage (Table 1). In line with previous reviews,^28,29^ a study with a score < 70% was considered to be of “high risk of bias” and a score of ≥ 70% was considered to be of “low risk of bias”.

**Table 1.** Appraisal of quality and risk of bias based on the CASP tool for cohort studies.

Risk of bias components	Bennett et al., 2015	Jacobsen et al., 2021	Jurdi et al., 2021	Korde et al., 2023	Manda et al., 2020	Mishra et al., 2021	Oswald et al., 2022	Tonino et al., 2019	Score %
A clear aim is identified (population, risk factors, outcomes considered)	1	1	1	1	1	1	1	1	100%
The recruitment of participants is acceptable (generalisability of the findings)	0	1	0	0	0	1	0	0	25%
The measurement of the exposure (objective, validated measurements)	1	1	1	1	1	1	1	1	100%
The accuracy of the outcome (objective measurements, statistical analysis)	1	1	1	1	0	1	1	0	75%
Confounding factors have been taken account in the analysis	0	0	0	0	0	1	0	0	12.5%
The confounding factors have been identified	1	0	0	0	0	1	0	1	37.5%
The follow-up of participants is complete (dropout rate, missing data)	1	1	0	0	0	1	0	0	37.5%
The follow-up of participants is long enough	0	1	0	1	0	1	1	0	50%
The credibility of the results (bias, design and methods)	1	1	1	1	1	1	1	1	100%
The generalisability of the results (cohort included, setting)	0	0	0	0	0	0	0	0	0%
Other available evidence, studies in support of the findings	1	1	1	0	1	1	1	1	87.5%
The implications of the study for practice are described	1	1	1	1	0	1	1	1	87.5%
Score (%)	67%	75%	50%	50%	33%	92%	58%	50%	

1 = yes, 0 = no or not sure. Score of ≥70% = low risk of bias, score of < 70% = high risk of bias.

### Data Extraction

The following data were extracted from each eligible study (Table 2): title, publication date, number of patients, design and study objectives. Information regarding study population was also extracted including age, gender and cancer type being MM. Intervention characteristics included study duration, follow-up and type of wearable device. Extracted data relating to adherence included the type of wearable, device outcome, time worn by patient and overall adherence where available. Device outcomes included data such as heart rate, physical activity and sleep. Patient-reported outcome measures such as quality of life, were also collected.

**Table 2.** Overview of studies using wearable technology including patients on treatment for multiple myeloma (MM).

Author, date and country	Sample characteristics	Study design Main objective(s)	Data collection tool(s)	Adherence	Duration of follow-up	Considerations of bias	Results
Bennett et al., 2016 (USA)	n = 32 MM: n = 14 HSCT	Prospective cohort study To explore relationship between physical activity and symptom burden	Fitbit ^TM^ Flex (daily steps) PRO-CTCAE (35 items) PROMIS Global- 10	No definition	3–4 weeks during hospitalisation + for 4 weeks after discharge = 8 weeks	mostly white participants (84%) mostly men (63%) 25% of those eligible enrolled 35% drop out rate	Reduced physical activity correlated with increased symptom burden but no correlation with quality of life Wearable devices are promising as an acceptable measure physical activity during treatment for MM
Hacker et al., 2022 (USA)	n = 32 MM: n= 32 High-dose CXT + HSCT	Randomized Clinical Trial To test the acceptability, feasibility, and preliminary effects of an activity intervention (STEPS) compared with usual care	Fitbit Alta (daily steps) Actiwatch Spectrum Pro EORTC QLQ-C30 PROMIS Chalder fatigue scale	Defined as >10 hours of use/day 76% of the participants wore the physical activity tracker device more than 90% of study days	Baseline (prior to hospitalisation lasting 2 weeks) + 7 weeks after discharge = 2 time points	mostly white participants (84%) mostly men (66%) 71% of those eligible enrolled 0% drop out rate	Reduced physical activity after HCT Wearable devices are acceptable and feasible to measure physical activity during treatment for MM
Jacobsen et al., 2021 (Germany)	n = 79 MM: n = 15 Monoclonal antibody, CAR-T, HSCT	Prospective cohort study To evaluate symptom-burden and feasibility of a wearable device to monitor physical activity in the inpatient and outpatient setting.	Everion, Biovotion AG (average steps/day) - PRO (unvalidated) (4 items)	Defined as >10 hours of use/day Adherence of 83.0% (inpatient) vs 89.6% (outpatient)	Inpatients: 8-59 days (max 50 days) = 7 weeks Outpatients: max 30 days = 4 weeks	ethnicity not reported but likely mostly white mostly men (56%) 81% of those eligible enrolled 35% drop out rate inpatients 12% drop out outpatients 2 different cohorts but only descriptive statistics applied PRO not validated	Reduced physical activity in inpatients compared to outpatients Use of wearable device to monitor physical activity was demonstrated to be feasible in both inpatient and outpatient setting
Jurdi et al., 2021 (USA)	n = 63 MM: n = 21 HSCT	Prospective cohort study To assess the feasibility wearable device to measure physical activity and sleep trends and correlate with transplant-related outcomes.	Fitbit HR (average steps)	No definition of adherence Feasibility: cut-off of 70% of patients used the Fitbit HR throughout the hospitalisation.	Followed up during hospitalisation: 12-63 days.	mostly white participants (87%) men (51%) unable to assess how many patents were eligible. 14% drop out rate comparison of autologous and allogeneic HCT groups	Reduced physical activity in both groups but more in allogenic HSCT Physical activity was not associated with HSCT-related outcomes Wearable devices are acceptable to measure physical activity during treatment for MM
Korde et al., 2023 (USA)	n = 40 MM: n = 40 2 cohorts: < 65y vs > 65y Proteasome inhibitors, immunomodulatory agents, monoclonal antibody, chemotherapy & steroids.	Prospective cohort study To assess the feasibility of remote monitoring activity and its relationship with QOL.	Garmin Vivofit (average steps/24hr) EORTC QLQ-C30 EORTC QLQ-MY20	Defined as >13 patients in each 20-patient cohort compliant with capturing data for ≥16 h of a 24-hr period, in ≥60% of days of ≥4 induction cycles.	Baseline (1-7 days prior to chemotherapy initiation) + continuously for up to 6 cycles of chemotherapy. = 1 - 6 months	ethnicity not reported. men (50-55%) unable to assess how many patients were eligible but participants needed to pre-own compatible smart phones or tablets. 48% drop out rate	Feasibility of passive wearable monitoring was challenging. Participants compliant with continuous data capture (53%) Activity data associated with QOL. Patient activity increased over time
Manda et al., 2020 (USA)	n = 84 MM: n = 84 Immunotherapy	Non-randomised Trial To capture real-world patient experience	Garmin Vivofit 3 (activity and sleep) QLQ-C30 QLQ-MY20 TSQM-9	No definition of adherence	Baseline + continuously every 4 weeks. = median follow up 8 months	mostly white participants (73%) men (49%) unable to assess how many patients were eligible no dropout rate reported	Reported results were interim results. High Compliance with ePRO reporting ≥ 87% Levels of activity and sleep duration comparable to healthy adults.
Mishra et al., 2021 (USA)	n = 47 MM: n = 3 HSCT	Prospective Cohort Study Assess pre- and post-HCT physical function	ActiGraph GT3X (Activity level) 6MWT IPAQ FACT-BMT	No definition of adherence	Baseline + days 30, 90, and 180 = 4 time points Median follow-up for surviving patients = 54.5 months (range, 26.3–59.7) and all patients = 25.7 months (range, 0.6, 59.7).	ethnicity not reported men (66%) 112 screen failures for potentially eligible patients 6% drop out rate	Patients (n = 45, 96%) tolerated ActiGraph monitoring, with a median wear time of 165.4 h (range, 104.6–168 h). Reduced functional capacity of HSCT patients at baseline Limited correlation between objective and self-reported physical function HSCT patients overestimated their physical function
Oswald et al., 2022 (USA)	n = 12 MM: n = 7 CAR-T	Prospective Cohort Study To assess the feasibility and acceptability of collecting PROs and activity data	Fitbit Inspire 2 (Steps and Sleep) PROMIS-29 FACT-G7 PRO-CTCAE	Defined as participants wearing a Fitbit for >50% of study days.	Baseline/day of CAR-T infusion (day 0) through day 91 post infusion. PRO - enrolment, days 0–7, 14, 21, 30, 60 and 90.	Mostly white participants ( 83%) men (50%) 27% of those eligible enrolled. 33% drop out rate. Did not consider all possible data collected by the Fitbit trackers (e.g., heart rate)	Adherence was met with Fitbits worn for 85% of study days (928/1092 days worn) High rates of PRO completion (85%)
Tonino et al., 2019 (Holland)	n = 12 MM: n = 4 Red blood cell transfusions (n= 4) Proteasome inhibitors (n= 4) Immunotherapy (n= 4)	Prospective Cohort Study To evaluate patient experiences with the VitalPatch wearable sensor and to evaluate the usability of data generated by the physIQ accelerateIQ monitoring system	AccelerateIQ VitalPatch PRO 11 items (unvalidated)	No definition of adherence wearability was measured by a survey	VitalPatch sensor worn for a maximum of 12 days.	ethnicity not reported. men (83%) unable to assess eligibility. 33% drop out rate PRO not validated	PRO data indicated minimal impact on the patient’s life at day 8 compared to baseline. 3 patients withdrew from the study because of skin irritation. Nurses reported that the Vital Patch was easy to use.

### Data synthesis and analysis

Microsoft Excel software was used to extract, organise, and store the study data. Due to the diversity of adherence measures, type of wearable devices used, and data points collected, a meta-analysis was not possible to conduct. Therefore, a narrative synthesis was employed to analyse the studies, using descriptive statistics to illustrate and summarise the features of the data.

As this is a systematic review and there is no direct patient involvement, national or institutional approval was not required. The review protocol was registered on the International PROSPERO review database: crd.york.ac.uk/PROSPERO/display_record.php?RecordID=461800

## Results

### Study Selection

A total of 170 papers were retrieved from the initial database search; after the duplicates were removed, 154 titles and abstracts were evaluated (Figure 1). The full texts of 22 relevant papers were reviewed based on the inclusion and exclusion criteria. Finally, nine studies reporting on 220 patients with MM met the criteria and were included in this systematic review. General study characteristics are described below and presented in Table B. Table 3 highlights the data regarding the wearable devices used in the studies.

**Table 3.** Overview of studies: MM patients, Wearable devices and adherence.

**Author, date and country**	**Myeloma Patients (n= 220)**	**Wearable Device**	**Adherence definition**	**Adherence outcomes**
Bennett et al., 2016 (USA)	n = 14	- Fitbit Flex	No definition of adherence	Not reported
Hacker et al., 2022 (USA)	n= 32	- Fitbit Alta - Actiwatch Spectrum	Defined as >10 hours of use/day	76% of the participants wore the physical activity tracker device more than 90% of study days
Jacobsen et al., 2021 (Germany)	n = 15	- Everion, Biovotion AG	Defined as >10 hours of use/day	83.0% (inpatient) vs 89.6% (outpatient)
Jurdi et al., 2021 (USA)	n = 21	- Fitbit HR	No definition of adherence	76% completion rate for patients using the device throughout hospitalisation.
Korde et al., 2023 (USA)	n = 40	- Garmin Vivofit	Defined as >13 patients in each 20-patient cohort compliant with capturing data for ≥16 h of a 24-hr period, in ≥60% of days of ≥4 induction cycles.	60% of patients wore the device for at least one cycle.
Manda et al., 2020 (USA)	n = 84	- Garmin Vivofit 3	No definition of adherence	Not Reported
Mishra et al., 2021 (USA)	n = 3	- ActiGraph GT3X	No definition of adherence	96% of patients wore the device for the 7-day study period
Oswald et al., 2022 (USA)	n = 7	- Fitbit Inspire 2	Defined as participants wearing a Fitbit for >50% of study days.	Participants collectively wore the device for 85% of the study days
Tonino et al., 2019 (Holland)	n = 4	- VitalPatch	No definition of adherence Wearability was measured by a survey	66% of participants wore the device throughout the study period

### Study Characteristics

Among the nine studies, seven were conducted in the United States of America,^30–36^ and two in Europe.^37,38^ Eight were prospective cohort studies^30–33,35–38^ and one^34^ was a nonrandomized controlled trial. The studies included in this review had various intervention periods ranging from 7 days to 25 weeks.

### Characteristics of Research Participants

Six studies included patients diagnosed with different types of haematological malignancy (ALL, AML, CML, NHL, Hodgkin’s lymphoma, CLL, DLBCL, and MDS), including 49 patients with MM.^30,31,35–38^ Three studies focused only on patients with MM, and included 156 patients.^31,32,34^ The number of participants in each study ranged from 12 to 84, and their age range from 21 to 82 years; however, the specific age ranges for patients with MM were not reported. A total of 220 patients with MM were included. Of the nine studies, three focused on patients undergoing chemotherapy,^33–35^ four looked at patients undergoing HCT,^30,31,33,36^ one explored patients undergoing CAR-T^35^ and one reported on patients undergoing various infusions^38^ (red blood cells, and intravenous chemotherapy and immunotherapy).

### Patient withdrawal due to wearable device

Of the nine studies, four reported patient withdrawal rates alongside the reasons for the withdrawal.^32,33,37,38^ Across these studies, a total of 33 (8.7%) patients withdrew due to reasons related to the wearable device, including skin irritation (n=3), band rash (n=1), discomfort (n=6), and device issues (n=10).

### Measurement Tools

The studies included in this review used various measurement tools (Table 3). Wearable devices were produced by six different companies: Fitbit (four studies^30,31,33,35^) Garmin (two studies^32,34^), Biovotion AG (one study^37^), VitalPatch (one study^38^), Actiwatch (one study^31^) and ActiGraph (one study^36^), and were worn on either the wrist (seven studies^30–36^), upper arm (one study^37^) or chest (one study^38^), for different time periods. All the nine studies used triaxial accelerometer devices which paired with either smart phone applications which uploaded data to a third-party platform, or directly to the platform via mobile data.

In six studies^30–32,34–36^ various patient-reported outcome measures and questionnaires were used (EORTC QLQ-C30, QLQ-MY20, FACT-G7, FACT-G and PROMIS) alongside the wearable technology, and two studies^36,37^ utilised unvalidated tools. Physical activity measures were primary outcomes in six studies,^30–33,35,37^ with changes in physical activity being evaluated through various means including wearable device data, PROs, and walking tests. Two studies^34,35^ also reported sleep data.

Of the nine studies, four evaluated the correlation between activity data and objective outcomes.^30,32,33,36^ Two studies found significant correlations between activity data and symptom burden,^30,32^ with a reduction in the number of daily steps being associated with fatigue, pain, physical function, and sleep. However, one study added that the correlation between physical activity and increased symptom burden did not bear any correlation with quality of life.^30^ Both studies estimated the correlation between PRO scores and physical activity using a linear mixed-effect model, and the Wald-test was used to compute p-values for the significance of association. Only one study examined patients solely with MM^32^; it observed positive correlations between improved physical function ePRO scores and increased patient activity across both cohorts (patients <65 years and ≥65 years).

Improvements with patient activity were correlated with those in EORTC QLQ-C30 physical functioning scores (p < 0.0001), increasing global health status scores (p = 0.02), and decreasing disease burden scores (p = 0.042). However, no association was seen between activity levels and future perspective and self-body image. One study^33^ found no association between physical activity and clinical outcomes such as neutrophil engraftment or length of hospital admission. However, one study^36^ showed that changes in a 6-minute walking test at day 30 following HSCT and changes from baseline had meaningful association with subsequent overall survival and non-relapsed mortality.

### Adherence

Of the nine studies in this review, seven^30–35,37^ evaluated the adherence of participants to wearing the device for a specific period, which ranged from 76% to 90%. However, these percentages were dependent on how each study defined “successful” adherence (Table 3).

Five reports^31–33,35,37^ examined adherence in terms of the completeness of the data collection, which were measured in a variety of ways, including wearing of device for a percentage of study days, a percentage of hours worn per study day, or a combination of percentage of hours over a percentage of study days.

Five papers^31–33,35,37^ reported the number of days of activity data required for a patient to be considered adherent, ranging from 50% to 80% of patients having recordable data available from between 5 and 90 consecutive days/nights. Four studies^31,33,35,37^ met their endpoints and concluded that adherence was acceptable to measure physical activity during treatment for MM. Only one^31^ of these studies looked exclusively at patients with MM. One study^32^, also addressing only patients with MM, found that adherence was challenging and did not meet its pre-defined end point. Two trials^31,37^ also defined adherence based on device wearing time (the minimum number of hours of use per day), with both studies using >10 hours/day. Overall adherence ranged from 50% to 90%, with 6 studies (67%) reporting it to be greater than 75%.

One report^38^ explored the wearability and usability of a wearable device using patient surveys, and one^36^ did not report any data regarding adherence. Of the seven studies that evaluated adherence, six^30,31,33–35,37^ met their endpoint and determined that that adherence was satisfactory. One study^32^ did not meet its endpoint.

## Discussion

This is the first systematic review to assess acceptability of using wearable devices to monitor physical activity in patients with MM. It highlights the limited number of studies which report on using wearable devices in patients with haematological malignancies, and specifically MM. The search process also highlighted four on-going studies^39–42^ with published abstracts which are exploring the use of wearable devices in patients with MM, for which the full papers had not been published at the time of submission of this review. This indicates a growing interest in understanding the use of wearable devices within clinical settings and, when published, their results will add to this review in better understanding the broader picture of this area.

The study outcomes of adherence were not dependent on or affected by the individual sample sizes or duration of the interventions, with results differing across the nine studies. The main impact of sample size or intervention is seen in the assessment of each study’s quality. The sample sizes and duration of intervention seen in this review were comparable to a systematic review^43^ of 25 studies looking at wearable devices in oncology, which found that sample sizes varied from 7 to 180 patients, and the duration of intervention ranged from 3 days to 38.5 weeks. This lack of consistency in the approach to researching wearable devices in both oncology and haemato-oncology means that results are difficult to generalise or translate effectively into clinical practice.

This review noted that wearable devices were most frequently used to objectively measure physical activity, as either duration of activity or step count. However, there is limited evidence regarding activity levels and correlation with patient outcomes. Four studies evaluated the correlation between objective physical activity data and PROs, finding some important correlations between the two outcome measures: a reduction in physical activity correlated with an increased symptom burden. However, only one study^32^ looked solely at patients with MM (n= 40) and found that there was significant correlation between the two. Only 26% (n= 37) of patients on the remaining three studies had MM, with the patients having a mixture of various haematological malignancies, and two of these studies found no significant correlation between PROs and activity. This suggests that there could be a correlation between activity and PROs in patients with MM that is not seen in those with different haematological malignancies, and that this type of research in the future can offer significant information towards better understanding and even predicting, quality of life for patients with MM using wearable devices.

There was also a lack of consistency in- the type of wearable device used across the included studies, the location of placement and even the purpose of the technology. This is comparable to a systematic review of wearable devices in oncology^44^ where it was found that five different brands were used including ActiGraph (71 studies; 36%), Fitbit (37 studies; 19%), Garmin (13 studies; 7%), and ActivPAL (11 studies; 6%). None of the studies explore how often the devices were charged or how long patients left them off during charging or other activities such as bathing, all of which could affect data accuracy and study outcomes. Although this is disappointing for researchers and HCPs, it also highlights where research is needed. Questions arise about which device is best to use, whether all researchers should use the same device across studies, or whether mobile telephone technology would allow for better real world data collection and easier movement into standard of care.

One study^36^ noted that a decline in a participant’s walking distance over time, correlated with a decrease in overall survival and non-relapsed mortality, which is similar to findings in multiple studies [44-46] and a meta-analysis [47] of patients with solid cancers ,which found that a lower risk of mortality was significantly associated with increased levels of physical activity. However, the study in this review only contained three patients with MM, and so conclusions on any associations in this patient group are difficult, and further research is required.

The overall adherence seen in this review (50% to 90%) is similar to a systematic review of 86 studies undertaken in oncology [48,49], where adherence ranged from 40% to 100%, with 73% of studies reporting adherence above 80%. However, of the studies that defined adherence only two^31,32^ looked at participants exclusively with MM, and these two had differing results regarding adherence (53 versus 76%). A study [49] which looked at wearable devices in the field of rehabilitation found >80% adherence in 76% (16) of patients, with five of those patients having MM. This suggests patients can and do adhere to using wearable devices, but further studies of participants solely with MM are warranted to explore adherence in greater detail.

Researchers face difficulty defining adherence levels using factors such as wear time per day or percentage of days worn. This was a similar finding in a general oncology setting^43^ where a definition of adherence was equally missing, and it was deemed crucial to resolve if we want to conclude anything from the data in wearable device studies. There is no consistency in the literature, and this will need to be addressed in future trials, if devices and approaches are to be validated for clinical practice.

No studies were conducted in developing countries. This is likely due to the limited availability of wearable devices or similar technology in those countries, which poses several challenges and disparities in healthcare access and outcomes [50]. Wearable devices and the associated technology can be prohibitively expensive for many patients with poor digital literacy in developed, but especially in developing countries, which alongside the requirement for reliable internet connectivity and smartphone access, highlights important issues in ensuring that all patients can benefit from advancements in health care technology [51].

This review has seen that the wearable device itself has caused patients to withdraw from study participation, due to skin irritation, general discomfort, and technical issues with the device. Studies have seen dropout rates ranging from 2.5-25% due to these reasons, which is something that must be considered when planning future research studies, and before incorporating any wearable device into practice. However, the largely high adherence rates seen, alongside the benefits of objective data collection, suggest that wearable devices could be an effective way of measuring physical activity when correctly monitored.

Whilst wearable devices offer promising potential for enhancing patient care, it is important to note that current evidence base does not conclusively support their actual value to patients or care providers. Most of the studies on wearable devices in patients with MM are short term observational and involve small sample sizes, limiting the generalisation of their findings. Therefore, comparative longitudinal studies that evaluate wearable devices against other forms of healthcare interventions, such as educational programs, could provide insights into the relative value of wearable devices and help understand any long-term impacts or potential limitations.

Another limitation is the exclusion of patients who have either not undergone or have already completed treatment, commonly referred to as survivorship. By not including the experiences of these individuals, the generalisation and applicability of the findings could be limited.

### Conclusion

Although wearable technology is being used widely in oncology, there appears to be a scarceness of research in patients with MM, a condition known to directly affect performance status. This review has reported significant heterogeneity in the measured outcomes, duration of intervention and type of device used. It has found that adherence to wearable devices varies from 50% to 90%, suggesting patients’ good acceptance of the technology, and an objective method of monitoring physical activity in patients with MM in future research studies. However, there is heterogeneity in definitions of adherence criteria.

Wearable devices are important as they provide continuous monitoring of various health metrics such as heart rate, activity levels, sleep patterns, and more, which can be particularly valuable for early detection of health issues, allowing for timely intervention. The continuous data collected by wearable devices can help healthcare providers tailor treatments to individual patients, improving the efficacy of treatments and reducing adverse effects. These devices can also empower patients by giving them real-time feedback on their health and potentially encourage healthier behaviours. Monitoring patients with a wearable device, alongside traditional methods of review, may be an effective way to evaluate their overall health and wellbeing. As there is currently no cure for MM, we must explore all avenues on how to help our patients improve their quality of life, and wearable technology is one of those.

A consensus is required on the methodology of measuring activity using wearable devices, and we should attempt to explore and validate a method in our MM patient population. Looking forward, we need to study the association between the data collected by wearable devices and clinical outcomes, exploring the objectively measured physical activity data collected and their ability to predict relevant clinical outcomes.

### Implications for Practice

Wearable fitness devices seem to be an accepted form of monitoring physical activity in patients receiving treatment for MM, and can provide objective data capture, which can be used alongside current practice.

**Table:** Abbreviations

6MWT - 6 min walk test PS - Performance Status CAR-T - Chimeric antigen receptor therapy CXT - Chemotherapy FACT-BMT - Functional Assessment of Cancer Therapy - Bone Marrow Transplant FACT-G - Functional Assessment of Cancer Therapy – General FACT-G7 - Functional Assessment of Cancer Therapy - General 7 item version HSCT - Haematopoietic stem cell transplantation IPAQ - International physical activity questionnaire PRO-CTCAE - Patient-Reported Outcomes version of the Common Terminology Criteria for Adverse Events QOL – Quality of Life	MM – Multiple Myeloma NDMM – Newly Diagnosed Multiple Myeloma ASCT - Autologous Stem Cell Transplant PRO – Patient Reported Outcome PROMIS-29 - Profile Physical and Mental Health Summary Scores PROMIS Global- 10 - Patient-Reported Outcomes Measurement Information System – Global Health Score EORTC - European Organisation for Research And Treatment Of Cancer QLQ-C30 - Quality of Life questionnaire – Core 30 questions QLQ-MY20 – Quality of Life questionnaire – Multiple Myeloma Module TSQM-9 - Treatment Satisfaction Questionnaire for Medication – Version 9

### Authors’ Contribution

Conceptualization: Tommy Brown, Ann Muls; Methodology: Tommy Brown, Ann Muls; Formal analysis and investigation: Tommy Brown, Ann Muls; Writing - original draft preparation: Tommy Brown, Ann Muls; Writing - review and editing: Tommy Brown, Ann Muls, Charlotte Pawlyn, Kevin Boyd, Susanne Cruickshank; Funding acquisition: Tommy Brown; Resources: Tommy Brown, Ann Muls.

### Competing of Interest – COPE

Tommy Brown - Research Support: Fitbit Inc. The other authors declare that they have no conflict of interest.

### Ethical Conduct Approval

Not required for this systematic review

### Informed Consent Statement

All authors and institutions have confirmed this manuscript for publication.

### Data Availability Statement

All are available upon reasonable request.

## Supplementary Material

2325639 Appendix 1 Appendix 1 - Search Strategy
