# Supplementary material for: The acceptability of using wearable electronic devices to monitor physical activity of patients with Multiple Myeloma undergoing treatment: a systematic review
Source: Clin Hematol Int. 2024 Jul 29;6(3):38–53. doi: 10.46989/001c.121406 (PMC11391912; doi:10.46989/001c.121406)
Supplement: 2325639 Appendix 1 — Appendix 1 - Search Strategy [file chi_2024_6_3_121406_236984.pdf]

## Appendix 1 - Search Strategy

Embase <1974 to 2023 September 06>

Ovid MEDLINE(R) ALL <1946 to September 06, 2023>

Global Health <1973 to 2023 Week 35>

Journals@Ovid Full Text <September 05, 2023>

1 "wearable electronic devices AND OR multiple myeloma patients".mp. [mp=ti, ab, hw, tn, ot, dm, mf, dv, kf, fx, dq, bt, nm, ox, px, rx, an, ui, sy, ux, mx, cw, tx, sh, ct] 0

2 wearable electronic devices, multiple myeloma patients.mp. [mp=ti, ab, hw, tn, ot, dm, mf, dv, kf, fx, dq, bt, nm, ox, px, rx, an, ui, sy, ux, mx, cw, tx, sh, ct] 0

3 wearable devices, haematology.mp. [mp=ti, ab, hw, tn, ot, dm, mf, dv, kf, fx, dq, bt, nm, ox, px, rx, an, ui, sy, ux, mx, cw, tx, sh, ct] 0

4 wearable, haematology.mp. [mp=ti, ab, hw, tn, ot, dm, mf, dv, kf, fx, dq, bt, nm, ox, px, rx, an, ui, sy, ux, mx, cw, tx, sh, ct] 0

5 wearable, hematology.mp. [mp=ti, ab, hw, tn, ot, dm, mf, dv, kf, fx, dq, bt, nm, ox, px, rx, an, ui, sy, ux, mx, cw, tx, sh, ct] 0

6 wearable device, Haematology*.mp. [mp=ti, ab, hw, tn, ot, dm, mf, dv, kf, fx, dq, bt, nm, ox, px, rx, an, ui, sy, ux, mx, cw, tx, sh, ct] 0

7 tracking device, patients.mp. [mp=ti, ab, hw, tn, ot, dm, mf, dv, kf, fx, dq, bt, nm, ox, px, rx, an, ui, sy, ux, mx, cw, tx, sh, ct] 13

8 wearable.mp. [mp=ti, ab, hw, tn, ot, dm, mf, dv, kf, fx, dq, bt, nm, ox, px, rx, an, ui, sy, ux, mx, cw, tx, sh, ct] 70935

9 myeloma.mp. [mp=ti, ab, hw, tn, ot, dm, mf, dv, kf, fx, dq, bt, nm, ox, px, rx, an, ui, sy, ux, mx, cw, tx, sh, ct] 275616

10 8 and 9 170

11 8 or 9 346381

12 remove duplicates from 10 154

13 10 and 11 170

14 remove duplicates from 13 154
